# Improving the Estimation of Subgroup Effects for Clinical Trial Participants with Multimorbidity by Incorporating Drug Class-Level Information in Bayesian Hierarchical Models: A Simulation Study

**DOI:** 10.1177/0272989X211029556

**Published:** 2021-08-18

**Authors:** Laurie J. Hannigan, David M. Phillippo, Peter Hanlon, Laura Moss, Elaine W. Butterly, Neil Hawkins, Sofia Dias, Nicky J. Welton, David A. McAllister

**Affiliations:** Nic Waals Institute, Lovisenberg Diaconal Hospital, Oslo, Norway; Population Health Sciences, Bristol Medical School, University of Bristol, Bristol, UK; Institute of Health and Wellbeing, University of Glasgow, Glasgow, UK; Population Health Sciences, Bristol Medical School, University of Bristol, Bristol, UK; Institute of Health and Wellbeing, University of Glasgow, Glasgow, UK; NHS Greater Glasgow & Clyde, UK; School of Medicine, University of Glasgow, Glasgow, UK; Institute of Health and Wellbeing, University of Glasgow, Glasgow, UK; Institute of Health and Wellbeing, University of Glasgow, Glasgow, UK; Centre for Reviews and Dissemination, University of York, York, North Yorkshire, UK; Population Health Sciences, Bristol Medical School, University of Bristol, Bristol, UK; Institute of Health and Wellbeing, University of Glasgow, Glasgow, UK

**Keywords:** hierarchical modeling, individual-patient data meta-analysis, medical ontologies, multimorbidity, subgroup analysis

## Abstract

**Background:**

There is limited guidance for using common drug therapies in the context of multimorbidity. In part, this is because their effectiveness for patients with specific comorbidities cannot easily be established using subgroup analyses in clinical trials. Here, we use simulations to explore the feasibility and implications of concurrently estimating effects of related drug treatments in patients with multimorbidity by partially pooling subgroup efficacy estimates across trials.

**Methods:**

We performed simulations based on the characteristics of 161 real clinical trials of noninsulin glucose-lowering drugs for diabetes, estimating subgroup effects for patients with a hypothetical comorbidity across related trials in different scenarios using Bayesian hierarchical generalized linear models. We structured models according to an established ontology—the World Health Organization Anatomic Chemical Therapeutic Classifications—allowing us to nest all trials within drugs and all drugs within anatomic chemical therapeutic classes, with effects partially pooled at each level of the hierarchy. In a range of scenarios, we compared the performance of this model to random effects meta-analyses of all drugs individually.

**Results:**

Hierarchical, ontology-based Bayesian models were unbiased and accurately recovered simulated comorbidity-drug interactions. Compared with single-drug meta-analyses, they offered a relative increase in precision of up to 250% in some scenarios because of information sharing across the hierarchy. Because of the relative precision of the approaches, a large proportion of small subgroup effects was detectable only using the hierarchical model.

**Conclusions:**

By assuming that similar drugs may have similar subgroup effects, Bayesian hierarchical models based on structures defined by existing ontologies can be used to improve the precision of treatment efficacy estimates in patients with multimorbidity, with potential implications for clinical decision making.

Multimorbidity, which is defined as the presence of 2 or more chronic conditions within an individual, is common and increasing. More than half of patients with any chronic disease have multimorbidity.^1^ This represents a challenge because the applicability of clinical trial results to patients with multimorbidity is uncertain. Consequently, several clinical guideline bodies have urged caution in applying trial results to patients with multimorbidity,^2^ while in practice, patients with multimorbidity are less likely to receive drug treatments shown to be effective in clinical trials, even where there is no contraindication to therapy.^3–6^

One reason for this uncertainty is that multimorbidity is underrepresented in clinical trials.^7,8^ For this reason, some researchers have used observational data—particularly administrative data, in which multimorbidity is common—to estimate treatment effects. However, such pharmacoepidemiological approaches are subject to confounding by indication^9^ despite methodological advances^10,11^ and so remain restricted in terms of their utility to support medical decision making in this regard.

Moreover, while multimorbidity may not be present at the same rate in clinical trials compared with in the community, it is nonetheless common. For half of 22 medical conditions, we found that at least one-third of trial participants in standard industry-funded clinical trials had multimorbidity. Furthermore, similar comorbidities were common in the trial and community settings.^8^ Consequently, there is both a need and an opportunity to determine whether treatment effects in clinical trials differ for subgroups of patients with and without multimorbidity and for different patterns of multimorbidity.

Reliably estimating treatment effects for subgroups in individual clinical trials is notoriously difficult.^12–14^ Claims of subgroup effects made in clinical trial reports are frequently unsupported by appropriate statistical evidence.^15^ While prespecified subgroup analyses can be adequately powered, there are often insufficient numbers of participants to estimate differences in effects across subgroups (especially for specific comorbidities) with adequate precision to inform clinical decision making.^13^ Moreover, simple methods to reduce the risk of false positives (i.e., asserting that there is heterogeneity when none exists) do so at the expense of precision and increase in type 2 errors.^16^ Consequently, attempts to estimate treatment effects for patients with multimorbidity are likely to suffer from both poor sensitivity and poor specificity.

Meta-analyses pool findings across trials to improve precision,^17^ and individual patient data (IPD) meta-analyses can be used to pool treatment effect estimates for participants with specific characteristics such as particular comorbidities.^18^ Even for meta-analyses, however, estimating subgroup effects with sufficient precision to inform clinical decision making is challenging because, compared with the overall trial, data on particular subgroups can be limited.

One approach to dealing with limited data is to use hierarchical modeling.^19^ Within a Bayesian framework, hierarchical modeling is straightforward^20^ and has previously been shown to be useful for analyzing clinical trial data. Examples include performing subgroup analyses^19^ and estimating adverse treatment effects.^21^ Such approaches rely on the assumption that information can be shared between parameters. In an information-sharing approach to subgroup analysis, Dixon and Simon^20^ assumed that treatment-covariate interactions came from a common prior distribution. Similarly, in estimating effects of treatments on adverse events, Berry and Berry^21^ assumed that events occurring within specific body systems (e.g., the gastrointestinal system) were related. In both examples, separate estimates were “partially pooled,” increasing precision and attenuating extreme values toward the group-level mean (shrinkage). Partial pooling and shrinkage are established features of hierarchical models.^22^ These are desirable features for subgroup analyses as, where the assumption that information can be shared holds, they are likely to improve our ability to detect true subgroup effects while reducing false positives.

Despite these desirable properties, the use of hierarchical modeling in subgroup analyses has thus far has been limited. One reason for the limited adoption may be uncertainty in how to allow sharing of information between different trials, that is, how should hierarchical models be structured. Using established drug-related ontologies such as the World Health Organization Anatomic Chemical Therapeutic Classifications (WHO-ATC), which is a treelike classification scheme based on therapeutic indications and chemical forms,^23^ and MED-RT, a US-based ontology that provides finer granularity for mechanisms of action,^24^ may help overcome this barrier. Such ontologies represent expert knowledge about similarities, differences, and relationships between different drugs in terms of indications, chemical structures, and other features, providing a starting point from which to define a hierarchical structure for modelling.

In other fields, relationships within ontologies have been used to predict protein–protein interactions, diagnoses, and the classification of chemicals.^25^ Ontologies have also been exploited to support the management and execution of clinical trials.^26–29^ Because WHO-ATC, MED-RT, and other ontologies are publicly available, they provide a transparent starting point for analyses. This aspect of ontologies is appealing in the field of clinical trial meta-analysis where transparency, consistency, and prespecification are highly prized.^30^

## The Current Study

**Figure 1:**
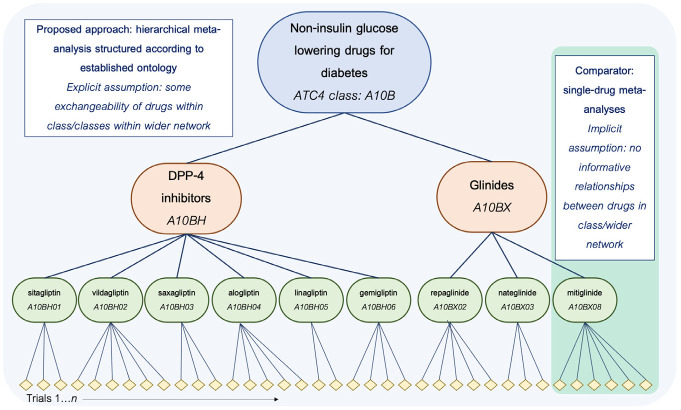
Schematic overview of the proposed and comparator approaches in the current simulation study, shown for drugs in 2 classes (A10BH and A10BX) within the wider grouping (all A10B drugs) used as the basis for the simulation. Note that only a subset of the hierarchy is shown in the interest of managing space constraints; in the study, the full hierarchical meta-analytic model is applied to a network incorporating all A10B drugs, and single-drug meta-analyses are similarly run for all drugs in the network.

In this study, we address the question of whether partial pooling of subgroup effects in existing clinical trial data, using structures borrowed from established drug classification ontologies, is feasible and has the potential to support clinical decision making. To do this, we first simulate data sets with interactions between a group of noninsulin glucose-lowering drugs for diabetes and a single hypothetical comorbidity, based on the characteristics of real trials. Next, we apply Bayesian hierarchical generalized linear models, with individual trials nested within drugs nested within ATC drug classes, to these data. Our use of an established ontology to structure a hierarchical meta-analysis is based on the simple assumption that drugs that are similar may behave similarly in subgroups. We compare the performance of these ontology-based hierarchical models, in terms of their recovery of comorbidity-treatment interaction effects for individual drugs, with that of standard, single-drug meta-analyses (see Figure 1 for an overview). In addition, we highlight specific properties of these models that emphasize their potential utility in IPD meta-analyses of comorbidity-based subgroup effects within clinical trial data.

## Methods

### Identification and Classification of Existing Trials as the Basis for Simulation

We opted to base our simulations on the characteristics of real trials of an exemplar drug grouping: noninsulin glucose-lowering drugs for diabetes. We identified all relevant existing trials on the US clinical trials register (clinicaltrials.gov) that met a set of prespecified selection criteria (Prospero protocol CRD42018048202^31^). Briefly, these included a minimum enrollment of 300 participants; a start date of January 1, 1990, or later; being a phase 2/3, 3, or 4 trial; and having an upper age limit of 60 y or older. We used trial-level descriptive information that is publicly available on their clinicaltrials.gov record trials to define the structure of the simulated data to reflect, as closely as possible, the characteristics of a real IPD that is (theoretically) available from trial sponsors. Specifically, we obtained information about the number of trials available per drug and class and the number of participants enrolled in each trial, and we used these characteristics as the basis of our simulations of subgroup effects for each trial. For simplicity, all trials were treated as single-arm versus placebo/usual care in the simulation.

After classification according to the WHO-ATC ontology, we included 161 trials involving 210,046 participants, of 24 separate drugs from 7 different WHO-ATC 5-level classes (e.g., DPP-4 inhibitors, SGLT-2 inhibitors). Full details of the classifications are provided in Supplementary Appendix 1 and Supplementary Table S1.

### Data-Generation Procedure

We simulated data to generate trial-level subgroup effects for each of the 161 trials. This was to reflect a situation in which individual patient-level data for these trials had been shared and the effect of an interaction between a particular comorbidity and the drug treatment under investigation had been estimated for each trial in preparation for meta-analysis, which is a common analytical approach in IPD meta-analysis.^17^

Data were simulated based on an overall comorbidity-treatment interaction of −0.1 standard deviations at the level of the wider drug grouping (i.e., the top level of the hierarchy, reflecting the average interaction effect across all drugs). This was chosen as a minimum difference, which might plausibly be important for decision making, recognizing that subgroup interactions are likely to be modest in real applications. This effect size would mean that, for a treatment minus control arm difference in efficacy of 0.2 standard deviations, the treatment efficacy in patients with multimorbidity would be 0.1 standard deviations.

Trial-level effects were simulated by adding random variation around the overall comorbidity-treatment interaction effect at each level of the hierarchy (i.e., at the level of drug class, drug, and trial). We simulated 1000 data sets for each of a range of scenarios, reflecting different degrees of between-trial, between-drug, and between-class variability:

#### All levels: low variation

In this scenario, we simulated 1000 data sets with trial-level interaction effects by adding random variation of 0.05 SDs at the levels of drug class, drug, and trial to the fixed overall effect of −0.10 SDs. Data sets in this scenario represent situations in which all trials of a given drug, all drugs in a given class, and all classes of drugs in the hierarchy have highly similar estimates for a given comorbidity-treatment interaction effect.

#### All levels: medium variation

In this scenario, we simulated 1000 data sets with trial-level interaction effects by adding random variation of 0.15 SDs at the level of drug class, drug, and trial to the fixed overall effect of −0.1 SDs. Data sets in this scenario represent situations in which all trials of a given drug, all drugs in a given class, and all classes of drugs in the hierarchy have moderately similar estimates for a given comorbidity-treatment interaction effect.

#### All levels: high variation

In this scenario, we simulated 1000 data sets with trial-level interaction effects by adding random variation of 0.25 SDs at the level of drug class, drug, and trial to the fixed overall effect of −0.1 SDs. Data sets in this scenario represent situations in which all trials of a given drug, all drugs in a given class, and all classes of drugs in the hierarchy have relatively dissimilar estimates for a given comorbidity-treatment interaction effect.

#### Other scenarios: variation manipulated at a specific level of the hierarchy

We additionally simulated sets of 1000 data sets in scenarios in which, during the data-generation procedure, we manipulated variation at each level of the hierarchy in turn while keeping variation at the other levels constant at 0.05 SDs. So, for example, this allowed us to represent situations in which trial-level estimates of comorbidity-treatment interactions for a given drug were highly dissimilar, but drugs and drug classes behaved more consistently (i.e., a “trial-level: high variation” scenario, in which trial-level interaction effects were simulated by adding random variation of 0.05 SDs at the level of drug class and drug, but 0.25 SDs at the level of trial, to the fixed overall effect of −0.1 standard deviations).

In the main analyses, we assumed the prevalence of the comorbidity that defines the subgroup to be 20%. This value is used in determining the precision of the simulated trial-level interaction estimate, which is also based on the number of individuals enrolled in the trial and is the same across data sets and scenarios. Further details of the simulation procedure are given in Supplementary Appendix 2, and an abbreviated example of a simulated data set is presented in Supplementary Table S2.

### Modeling

To each simulated data set, we fitted 1) a hierarchical generalized linear model with all trials nested within drugs, nested within ATC-5 drug classes (henceforth “the full model”), and 2) hierarchical generalized linear models for all trials of each of the 24 drugs (henceforth “single-drug models”). We fit these models using the R-INLA package.^32^ Although integrated nested Laplacian approximation (INLA) performs approximate Bayesian inference, and offers less flexibility than software which uses Markov-chain Monte Carlo (MCMC) methods to fit models (specifically, hyperpriors must be Gaussian – an acceptable restriction in this case), model-fitting using R-INLA is very rapid, and gave good agreement to MCMC. Using R-INLA meant we could run the models on a larger number of simulated iterations and scenarios.

### Full Model Description

Interactions at the various levels of the hierarchy were specified as follows:

Trial-specific comorbidity-treatment interactions:

$$y_{z,d,c}\sim N(\mu_{z,d,c},s_{z,d,c}^{2})$$

Between-trial variation in comorbidity-treatment interactions:

$$\mu_{z,d,c}\sim N(\beta_{d,c},\tau_{d,c}^{2})$$

Between-drug variation in comorbidity-treatment interaction:

$$\beta_{d,c}\sim N(\gamma_{c},\sigma_{c}^{2})$$

Between-class comorbidity-treatment interaction:

$$\gamma_{c}\sim N(\alpha ,\zeta^{2})$$

The observed quantities *y* and *s* represent the comorbidity-treatment interaction and standard error at the level of the individual trial. Normal distributions are parameterized as mean and variance. The *z* subscript indicates the trial, the d subscript indicates the drug, and the c subscript indicates the drug class. The prior for the overall mean comorbidity-treatment interaction α was a normal distribution [ $N(0,2^{2})$ ]. This was chosen to correspond to the assumption that covariate treatment interactions are uncommon (when trial data are analyzed on an appropriate scale). For the between-drug class variation ζ , the between-drug (i.e., within-class) variation ( $\sigma_{c})$ , and the between-trial (i.e., within-drug) variation ( $\tau_{d,c}$ ) we, used half-normal priors on the standard deviations: $\;\mathrm{halfN}(0,1^{2})$ . The priors were selected to be relatively noninformative in relation to the values for variance at these levels used during the data generation (0.05^2^, 0.15^2^, 0.25^2^), to ensure that the performance of the full model was not artificially aided by our knowledge of these values.

Single-drug models were specified using the lowest 2 levels of the full model (i.e., trial specific and between trial) as outlined above and with the prior for the mean comorbidity-treatment interaction for a given drug $\beta_{d,c}$ parameterized as a normal distribution [ $N(0,2^{2})$ ].

### Performance Evaluation and Sensitivity Testing

We evaluated the performance of the full model against that of the single-drug models on their recovery of the drug-level interaction effect. In accordance with the framework outlined by Morris et al.,^33^ we compare the 2 approaches on several established performance measures: bias (the extent to which the effect is systematically over-/underestimated), mean squared error (MSE; the average extent to which the effect is over or underestimated) and root mean squared error (RMSE; equivalent to MSE but interpretable on the same scale as the data), change in precision relative to the single-drug model, and coverage (the proportion of credible intervals containing the true value). We used the R package *rsimsum*^34^ to derive estimates and Monte Carlo standard errors for each of the measures (RMSE was derived manually and standard errors approximated using the delta method).

To evaluate the sensitivity of the models to the prevalence of the subgroup-defining comorbidity, we reran all analyses with this value set at 10% and 50%, respectively.

The R code for the simulation and modeling is available at https://github.com/dmcalli2/simlt_interactions/blob/master/scripts/.

## Results

The relative performance of the full- and single-drug models is summarized in Table 1. Performance measures are aggregated across data sets and scenarios according to the amount of variability around the overall average interaction effect of −0.1 that was introduced at each level of the hierarchy during data generation, as described in the “Data-Generation Procedure” section above. As such, the top section of the table shows results for all data sets in 3 main scenarios: “all levels: low variation,”“all levels: medium variation,” and “all levels: high variation.” In the lower section of the table, the results are summarized for scenarios reflecting the effects of increasing variation at specific levels of the hierarchy.

**Table 1:** Summary of Performance Measures for Full- and Single-Drug-Only Models across All Simulated Data Sets for Different Scenarios^a^

Scenario			Single-Drug Model		Full Model	
Level(s)	Variation	Performance Measure	Estimate	MCSE	Estimate	MCSE
All	Low	Bias	0.013	0.000	0.001	0.000
All	Low	MSE	0.003	0.000	0.003	0.000
All	Low	RMSE	0.058	0.000	0.056	0.000
All	Low	Rel. prec.	—	—	89.004	1.415
All	Low	Coverage	0.968	0.001	0.852	0.002
All	Medium	Bias	0.012	0.001	0.000	0.001
All	Medium	MSE	0.025	0.000	0.028	0.000
All	Medium	RMSE	0.159	0.001	0.166	0.001
All	Medium	Rel. prec.	—	—	13.212	0.435
All	Medium	Coverage	0.812	0.003	0.674	0.003
All	High	Bias	0.010	0.002	−0.003	0.002
All	High	MSE	0.069	0.001	0.076	0.001
All	High	RMSE	0.263	0.001	0.276	0.001
All	High	Rel. prec.	—	—	3.952	0.287
All	High	Coverage	0.759	0.003	0.624	0.003
Trial	Medium	Bias	0.012	0.001	0.000	0.000
Trial	Medium	MSE	0.008	0.000	0.005	0.000
Trial	Medium	RMSE	0.091	0.000	0.068	0.000
Trial	Medium	Rel. prec.	—	—	127.704	1.767
Trial	Medium	Coverage	0.958	0.001	0.897	0.002
Trial	High	Bias	0.014	0.001	0.001	0.000
Trial	High	MSE	0.017	0.000	0.006	0.000
Trial	High	RMSE	0.132	0.001	0.077	0.000
Trial	High	Rel. prec.	—	—	251.074	2.848
Trial	High	Coverage	0.959	0.001	0.944	0.001
Drug	Medium	Bias	0.013	0.001	0.001	0.001
Drug	Medium	MSE	0.004	0.000	0.007	0.000
Drug	Medium	RMSE	0.064	0.000	0.082	0.001
Drug	Medium	Rel. prec.	—	—	34.080	0.558
Drug	Medium	Coverage	0.968	0.001	0.902	0.002
Drug	High	Bias	0.013	0.001	0.000	0.001
Drug	High	MSE	0.006	0.000	0.008	0.000
Drug	High	RMSE	0.077	0.000	0.090	0.001
Drug	High	Rel. prec.	—	—	10.278	0.274
Drug	High	Coverage	0.968	0.001	0.911	0.002
Class	Medium	Bias	0.013	0.001	0.001	0.001
Class	Medium	MSE	0.019	0.000	0.020	0.000
Class	Medium	RMSE	0.138	0.001	0.142	0.001
Class	Medium	Rel. prec.	—	—	3.648	0.443
Class	Medium	Coverage	0.785	0.003	0.542	0.003
Class	High	Bias	0.010	0.002	−0.003	0.002
Class	High	MSE	0.053	0.001	0.061	0.001
Class	High	RMSE	0.230	0.001	0.246	0.001
Class	High	Rel. prec.	—	—	−10.158	0.283
Class	High	Coverage	0.676	0.003	0.380	0.003

a See the “Data-Generation Procedure” subsection of the “Methods” section for a full definition of the scenarios. MSE, mean squared error; RMSE, root mean squared error; Rel. precision, percentage change in precision for full versus drug model; coverage, proportion of 95% credible intervals containing true effect; MCSE, Monte Carlo standard errors. RMSE estimates and corresponding MCSEs are not calculated by default in the *rsimsum* package and so are instead derived, with the MCSE approximated using the delta method, that is,

$SE_{\mathrm{RMSE}\;}=\;\sqrt{\frac{\mathrm{Var}\left(\sqrt{\mathrm{MSE}}\right)}{n}}\;\approx \;\sqrt{\frac{n{\left(SE_{\mathrm{MSE}}\right)}^{2}}{4n\;\times \;\hat{\mathrm{MSE}}}}=\;\frac{SE_{\mathrm{MSE}}}{2\;\times \;\surd \hat{\mathrm{MSE}}}$ .

**Figure 2:**
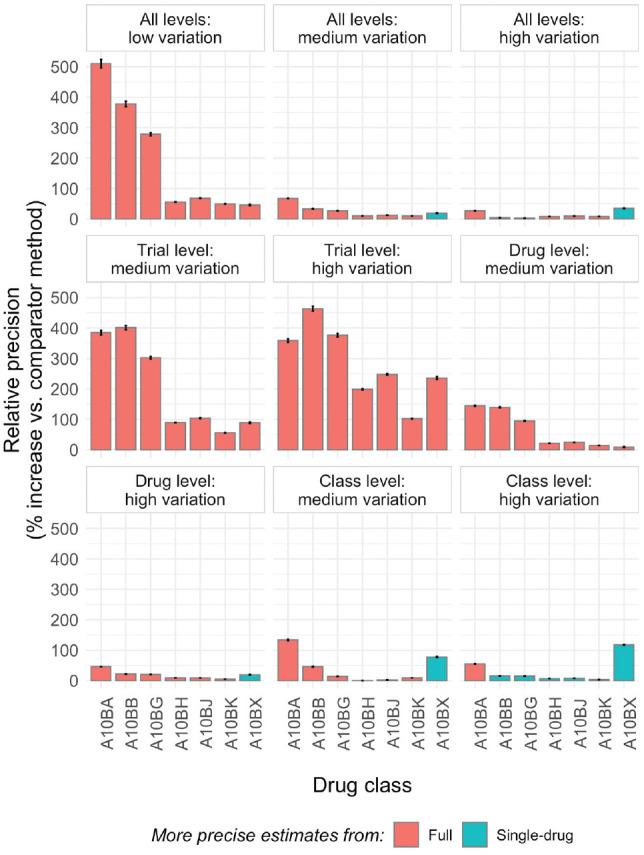
Summary of relative precision of drug level comorbidity-treatment interaction effects in full- versus single-drug model as a function of drug class. The error bars show the Monte Carlo standard errors; information on the number and size of trials for each drug class is found in Supplementary Table S1. In contrast to the values in Table 1, where relative precision is always displayed as the percentage change in precision for the full model relative to the drug model, here the “comparator method” is selected as whichever of the full or drug model is less precise to facilitate visual comparisons.

The full model estimated drug level comorbidity-treatment interaction effects without bias to the same extent as the drug-only models. MSE/RMSE values were similar in the full models and drug models in all cases, indicating that the degree of accuracy of the point estimates was at least equivalent. The largest difference in accuracy occurred in the “trial level: high variation” scenario, in which simulated trial-level effects were highly variable but drugs and drug classes relatively similar, when the full model was more precise by approximately 0.05 SDs (RMSE_drug_ = 0.13; RMSE_full_ = 0.08). The models differed more markedly on the other 2 measures of performance, precision and coverage, for related reasons. The full model estimated drug level comorbidity-treatment interactions, on average, more precisely in all scenarios, and substantially so in most cases. This is the expected result of information sharing at the level of drug class. The relative precision of estimates from the 2 approaches is illustrated, as a function of drug class, in Figure 2. Precision gains related to use of the full model are most substantial for drugs with a limited number of trials (or only small trials; see Supplementary Table S1 for drug-specific details) and when drugs and drug classes are more similar and trial-level estimates more varied (e.g., “trial level: high variation” scenario, middle panel). Precision in the full model was similar to or worse than in the drug model in all classes only when drug classes in the hierarchy were relatively dissimilar (e.g., “class level: high variation” scenario, bottom-right panel).

**Figure 3:**
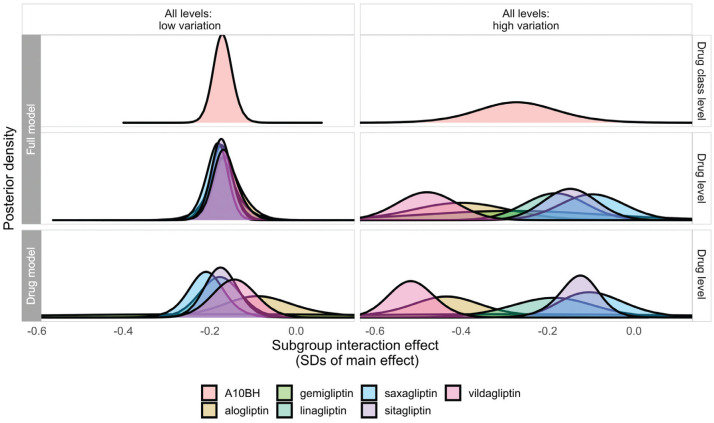
Posterior densities estimated for interaction effects at the drug class (top panel) and drug level (middle panel) from the full model and at the drug level (bottom panel) from single-drug models for drugs in the A10BH class in a single randomly selected data set in the “all levels: low variation” and “all levels: high variation” scenarios, illustrating properties of shrinkage at the drug level in the full model.

Coverage—the proportion of credible intervals including the “true” effect—was reduced in most instances in the full model, but this too is an expected feature of these models. It results from the combination of increased precision and shrinkage of extreme-for-class drug-level effect estimates toward the class average. These features are illustrated in Figure 3, which shows the posterior distributions of effects of drugs in a specific class, as estimated in the full model (middle panel) and single-drug models (lower panel), as they relate to the effect at the class-level effect (top panel). Drug-level effect distributions are shrunk (drawn toward the class-level mean) and estimated more precisely in the full model when drugs in the hierarchy are sufficiently similar (e.g., “all levels: low variation” scenario, left-hand panels of Figure 3). This means that the simulated effect for a given drug has a greater chance of falling outside the 95% credible intervals, but this is clearly desirable if the exchangeability assumption is met, as information from similar drugs has been used alongside the evidence available from trials of that drug to improve the estimate. In higher-variation scenarios, the extent to which drug-level estimates are influenced by class-level information is flexible and proportionate to the homogeneity of effects within the class. In the example shown in Figure 3 in the “all levels: high variation” scenario (right-hand panels), shrinkage is minimal, and only effects for gemigliptin and linagliptin are estimated more precisely in the full model, reflecting the fact that drugs and classes in this scenario are much less similar in terms of their subgroup effects.

**Figure 4:**
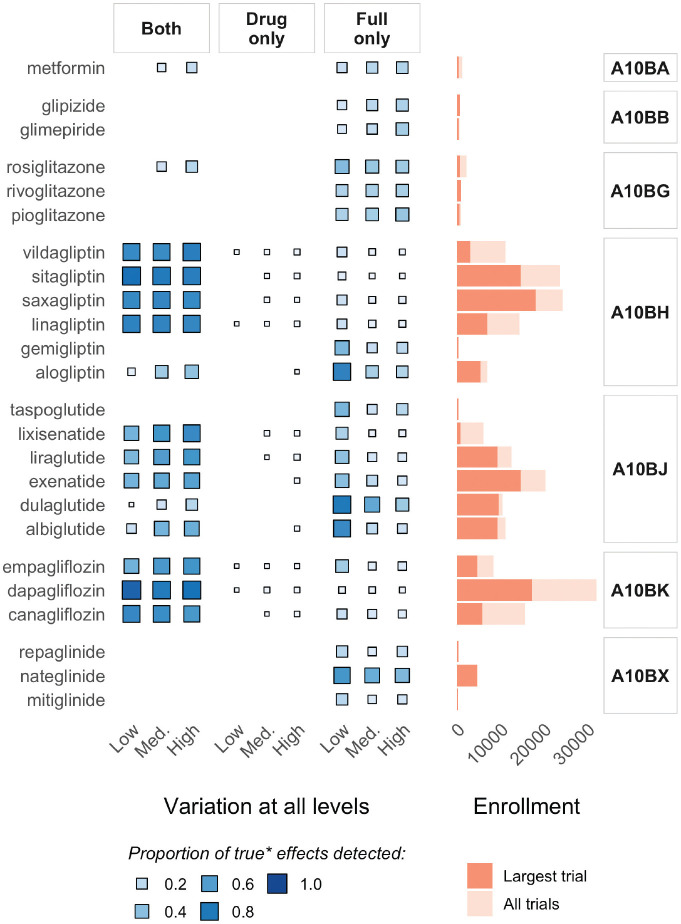
Illustration of the impact of increased precision in the full model: summarizing the proportion of all data sets with “true” effects in the 3 main scenarios in which credible intervals for the interaction effect estimate for each drug excluded zero (i.e., no interaction) in 1) both models, 2) the single-drug model only, and 3) the full model only, alongside enrollment information.

Figure 4 illustrates the potentially clinically meaningful impact of the increases in precision afforded by using the full model to estimate treatment interactions for related drugs in a hierarchy. It summarizes the proportion of all data sets in three main scenarios, in which a true drug-level subgroup effect of −0.10 or larger was able to be detected (i.e., with credible intervals not including zero) in 1) both models, 2) the single-drug model only, and 3) the full model only. For context, the panel on the right-hand side of the figure shows enrollment (i.e., *N*) for the largest trial per drug and aggregated across all trials of a drug. Effects in drugs with large trials and/or high aggregated enrollment were generally well detected in both models, although a substantial proportion was detected only in the full model, especially in the “all levels: low variation” scenario. The drug class–level information sharing in the full model was most beneficial for drugs with smaller/fewer trials (e.g., taspoglutide, and all drugs in classes A10BB and A10BX), in which true effects were detected only in the full model, regardless of the extent of variation in the hierarchy.

The results of the sensitivity analyses for different rates of subgroup-defining comorbidity prevalence are presented in the supplement (Supplementary Tables S3 and S4; Supplementary Figures 1–4).

## Discussion

In this article, we have demonstrated the feasibility of improving the estimation of treatment effects in subgroups by using Bayesian hierarchical meta-analytic models that share information across related trials based on established classification ontologies. Our simulations, based on characteristics of real trials of noninsulin glucose-lowering drugs for diabetes, show that partial pooling of subgroup effects across classes of drugs is 1) feasible, given the amount of data that is theoretically available from trial sponsors, and 2) effective at increasing the potential of subgroup effect estimation in the context of multimorbidity to influence clinical decision making.

In our simulations, Bayesian hierarchical models structured around the ATC ontology were unbiased and compared favorably to standard meta-analytic approaches in terms of both their precision (estimates are more precise) and conservatism (extreme estimates at drug and trial levels are shrunk toward class means) for estimating subgroup effects. Both of these represent nontrivial improvements in the estimation of drug-level subgroup effects, as a lack of data from individual trials/standard meta-analyses has typically meant that estimates are often too imprecise to be clinically useful, and concerns about false positives are commonly expressed in the literature around subgroups.^12,35^ More precisely and reliably estimated subgroup effects have greater potential to be incorporated into guidelines and influence clinical decision making. This is particularly important in the context of multimorbid patients, who represent more than 50% of individuals with any chronic condition, since current guidelines lack specific trial-based recommendations for the treatment of these individuals.^2^

**Figure 5:**
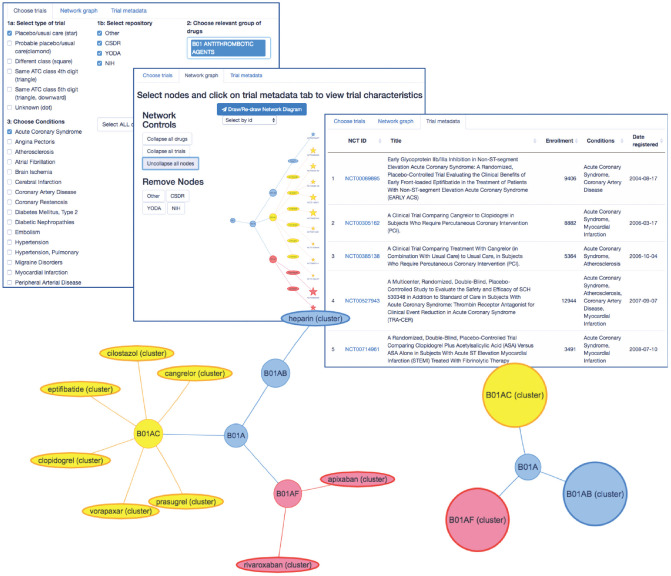
Introduction to an online tool (https://ihwph-hehta.shinyapps.io/duk_example_app/) for drawing network hierarchies of trials nested within drugs and drug World Health Organization Anatomic Chemical Therapeutic Classifications drug classes ascertained based on clinical trials with relevant meta-data on clinicaltrials.gov.

The core features of the model we have outlined will not be novel to anyone familiar with Bayesian hierarchical modeling and with concepts such as shrinkage and exchangeability. Such readers will also likely be aware of the difficulties inherent in formalizing prior knowledge for use in such models. We propose that the use of existing ontologies—specifically, although not exclusively, of drugs—such as the ATC system, to structure hierarchical models for meta-analyses of trial data is a widely applicable solution to this problem. In particular, we believe it to be immediately applicable to the challenge of estimating treatment effects in subgroups of patients likely to be poorly represented in clinical trials, such as those with specific comorbidities. To illustrate the portability of this approach and make it easier for others to use the WHO-ATC classification system, we have developed an online application (Figure 5) that can be used to visualize hierarchical classifications for a large set of trials registered in the clinicaltrials.gov database with relevant meta-data. The tool is available at https://ihwph-hehta.shinyapps.io/duk_example_app/. Users can select trial types, wider drug groupings, and conditions of interest to create a hierarchy that can then be visualized in different ways and for which the constituent trials (complete with NCT ID numbers) can be exported as a table. The tool can also be used to visualize networks of trials including drug-drug comparisons, and the principles of the model evaluated in this article can be straightforwardly extended to perform network meta-analyses including data from such trials. The R code for the diagram is also available https://github.com/dmcalli2/ctg_network_diagram. We anticipate continuing to update this tool using more recent data from clinicaltrials.gov.

An advantage of using existing ontologies such as the ATC system^23,24^ is that they have already codified a considerable body of expert knowledge about similarities/differences between different drugs. However, such ontologies might be used with modifications in real settings, in which a decision may be made to exclude a drug from a given class or exclude a class from the modeling. If, for example, a new class of drug were developed to address a perceived loss of efficacy in a particular subgroup (e.g., there is some evidence that certain classes of antiplatelet have a lower relative efficacy in women than in men^36^), it would not be appropriate to include other drug classes with the same physiological action within the modeling. Future work could also explore the use of more complex relationships between drugs by incorporating multiple ontologies.

### Limitations and Assumptions

The core assumption of this approach is that partially pooling interaction estimates across different drugs and, especially, across drug classes, is reasonable. When considering the validity of this assumption, it is worth taking into account the context for examining treatment effects in multimorbidity. In current practice, imprecise covariate-treatment interactions are typically either interpreted as evidence that no difference exists or as evidence that the treatment is not efficacious in patients with the multimorbidity, often according to some unstated prior belief. More precise estimates can be obtained from large observational data sets; however, such analyses are subject to confounding by indication, which has been called an “intractable” problem of epidemiology.^9^ Second, it is worth reiterating that the flexibility of these models means that hierarchical structures can be defined (and subsequently refined) based on expert opinion and empirical evidence regarding the validity of the core assumption for specific drug groupings. We anticipate that sensitivity analyses involving dropping specific classes and drugs from a hierarchy and comparing model fit will become a standard facet of this approach but acknowledge that further work is needed to develop formal assumption testing measures for use with real data. In particular, it will be important to develop contingencies to ascertain when a drug-level estimate is extreme because that drug truly behaves differently from others in its class and hence when the shrinkage afforded by a Bayesian hierarchical model is undesirable. Nonetheless, it should be borne in mind that an implicit assumption of single-drug meta-analyses is that drugs with similar mechanisms of action are no more likely to have similar subgroup effects associated with them than those operating via entirely different biological pathways. This assumption, were it to be made explicitly each time a single drug meta-analysis is performed, would likely be at least as debatable—if not more so—than the notion that related drugs may behave similarly to one another.

The scale of IPD sharing that is required for network meta-analyses is clearly greater than that which is needed for individual-drug meta-analyses. However, an important facet of the models we propose is that their benefits can be propagated to future work. Once a large network meta-analysis has been run, the posterior distributions of effects at drug class and drug levels can be used as priors in subsequent analyses. Indeed, as more trial sponsors provide access to individual-level participant data for increasing numbers of trials (e.g., via ClinicalStudyDataRequest.com^37^), it is possible to envisage the eventual compiling of a database of “off-the-shelf” priors for treatment-comorbidity interactions, which will enable health economists and others to more easily model the effect of treatments in people with multimorbidity.

The simulations in our study are subject to certain limitations. First, although we restricted our simulation to the trial level (rather than simulating IPD at the patient level) for computational reasons, we have only considered a situation in which IPD are available for all trials. That is, IPD would almost certainly be needed from all trials to obtain results stratified by particular comorbidities. To more pragmatically reflect the likely availability of data from clinical trials, it would be useful to explore models designed to accommodate aggregate data alongside IPD. However, one issue that this would exacerbate is the inconsistency of reporting of covariates. Given that covariate reporting is likely to be missing not at random, such models would need to account for bias or rely on specific covariate results being obtainable from sponsors (at an aggregate level) on request. Even within IPD, trials may not consistently record or define specific covariates, and the impact of these potential inconsistencies is not considered here. However, in the case of multimorbidity at least, we have recently demonstrated, using IPD for more than 100 trials shared by commercial sponsors, that it is possible to use generally well-recorded concomitant medication use data to facilitate the investigation of comorbidities.^8^ Second, for simplicity, we considered only a single comorbidity-treatment interaction. It would be useful in future studies to consider multiple comorbidities. This would mean simulating the impact of between-trial information sharing in models where there is also within-trial sharing via, for example, the Dixon-Simon model, in which a common prior is placed on all treatment-covariate interactions.^20^ Third, there is a range of important possible scenarios that we did not address in the current simulation. These include scenarios 1) with a smaller overall interaction effect (including no interaction) and 2) in which an interaction effect differs in magnitude or direction across classes within a hierarchy and scenarios in which comorbidities are absent in some trials. These (and many others) are relevant and realistic considerations for the challenges that real data may pose. However, the multiplicities created by so many possible scenarios are a limitation for all simulation studies, and the drawing up of bounds on the simulated universe(s) to be investigated is an inherent part of study design. In the future, potentially informed by the characteristics of real IPD where it is obtained, explorations of the capability of this approach to be informative in different scenarios would undoubtedly be beneficial. We have published our code, which we or others could modify to examine these and many other scenarios in future.

## Summary and Conclusions

Determining treatment effectiveness in multimorbidity is a challenging problem. If we are willing to assume—informed by existing ontologies—a level of similarity between drugs, hierarchical models can be used to estimate comorbidity-treatment interactions with improved precision. This has the potential to support trial-based decision making for patients with multimorbidity.

## Supplemental Material

sj-docx-1-mdm-10.1177_0272989X211029556 – Supplemental material for Improving the Estimation of Subgroup Effects for Clinical Trial Participants with Multimorbidity by Incorporating Drug Class-Level Information in Bayesian Hierarchical Models: A Simulation Study Supplemental material, sj-docx-1-mdm-10.1177_0272989X211029556 for Improving the Estimation of Subgroup Effects for Clinical Trial Participants with Multimorbidity by Incorporating Drug Class-Level Information in Bayesian Hierarchical Models: A Simulation Study by Laurie J. Hannigan, David M. Phillippo, Peter Hanlon, Laura Moss, Elaine W. Butterly, Neil Hawkins, Sofia Dias, Nicky J. Welton and David A. McAllister in Medical Decision Making
