# Supplementary material for: Improving the Estimation of Subgroup Effects for Clinical Trial Participants with Multimorbidity by Incorporating Drug Class-Level Information in Bayesian Hierarchical Models: A Simulation Study
Source: Med Decis Making. 2021 Aug 18;42(2):228–40. doi: 10.1177/0272989X211029556 (PMC8777306; doi:10.1177/0272989X211029556)
Supplement: sj-docx-1-mdm-10.1177_0272989X211029556 – Supplemental material for Improving the Estimation of Subgroup Effects for Clinical Trial Participants with Multimorbidity by Incorporating Drug Class-Level Information in Bayesian Hierarchical Models: A Simulation Study [file sj-docx-1-mdm-10.1177_0272989X211029556.docx]

# Improving the estimation of subgroup effects for clinical trial participants with multimorbidity by incorporating drug class-level information in Bayesian hierarchical models: a simulation study (Supplementary material)

[Improving the estimation of subgroup effects for clinical trial participants with multimorbidity by incorporating drug class-level information in Bayesian hierarchical models: a simulation study (Supplementary material)	1](#_Toc65264084)

[sAppendix 1	2](#_Toc65264085)

[sAppendix 2	2](#_Toc65264086)

[Supplementary references	2](#_Toc65264087)

[Supplementary tables	3](#_Toc65264088)

[Supplementary figures	9](#_Toc65264089)

[sTable 1. Classification of drugs from the diabetes trial-set into classes based on WHO-ATC-5](#_Toc73950501)

[sTable 2. Example dataset from simulation (abbreviated to 20 rows)](#_Toc73950502)

[sTable 3. Summary of performance measures for full and single drug only models across all simulated datasets for different scenarios – LOW SIMULATED COMORBIDITY PREVALENCE (10%)](#_Toc73950503)

[sTable 4 Summary of performance measures for full and single drug only models across all simulated datasets for different scenarios – HIGH SIMULATED COMORBIDITY PREVALENCE (50%)](#_Toc73950504)

sFigure 1 Summary of relative precision of drug level comorbidity-treatment interaction effects in full vs. single drug model as a function of drug class– LOW SIMULATED COMORBIDITY PREVALENCE (10%)

sFigure 2 Summary of relative precision of drug level comorbidity-treatment interaction effects in full vs. single drug model as a function of drug class– HIGH SIMULATED COMORBIDITY PREVALENCE (50%)

sFigure 3 Illustration of the impact of increased precision in the full model: summarising the proportion of all datasets with “true” effects in the three main scenarios in which credible intervals for the interaction effect estimate for each drug excluded zero (i.e., no interaction) in i) both models; ii) the single drug model only; and iii) the full model only, alongside enrollment information – LOW SIMULATED COMORBIDITY PREVALENCE (10%)

sFigure 4 Illustration of the impact of increased precision in the full model: summarising the proportion of all datasets with “true” effects in the three main scenarios in which credible intervals for the interaction effect estimate for each drug excluded zero (i.e., no interaction) in i) both models; ii) the single drug model only; and iii) the full model only, alongside enrollment information – HIGH SIMULATED COMORBIDITY PREVALENCE (50%)

# sAppendix 1

Each set of trials was organised into a hierarchy based on the biological mechanism of action of the drugs under study. For the diabetes trial-set, the WHO-ATC-5 level grouping of included drugs was closely reflective of the mechanism of action, so this was used as a node in the hierarchy. As a result, diabetes trials were nested within drugs, drugs were nested within WHO-ATC-5 drug classes, and drug classes were nested within the wider drug grouping (the WHO-ATC-4 level code A10B). The full network diagram for the diabetes trials can be viewed online here: <https://ihwph-hehta.shinyapps.io/duk_example_app/>. The classifications for the diabetes drugs are shown in sTable 1.

# sAppendix 2

In order to simulate interaction effects and their precision at the trial-level (but without simulating IPD in full), four groups per trial were defined overall: two for each arm (those with and without the comorbidity). We assumed one-to-one randomisation within a two-group parallel design, and that the comorbidity was equally common in the intervention and control arms. The precision of the comorbidity-treatment interaction estimate was approximated as the inverse of the sum of the standard errors-squared for each of the four groups, where the standard error for each group was estimated as the standard deviation for the standardised outcome (by definition one), divided by the square root of the number of participants in that group. The N-per-group was calculated by combining the trial-specific enrolment (as recorded in clinicaltrials.gov) and the specified prevalence for the comorbidity (see below).

An example dataset from a single iteration of the simulation for the diabetes trial-set is shown in sTable 3.

# Supplementary references

1.	Pathak J, Chute CG. Analyzing categorical information in two publicly available drug terminologies: RxNorm and NDF-RT. J Am Med Informatics Assoc. 2010;

2.	Iglay K, Hannachi H, Joseph Howie P, Xu J, Li X, Engel SS, et al. Prevalence and co-prevalence of comorbidities among patients with type 2 diabetes mellitus. Curr Med Res Opin. 2016;32(7):1243–52.

3.	Matcham F, Rayner L, Steer S, Hotopf M. The prevalence of depression in rheumatoid arthritis: a systematic review and meta-analysis. Rheumatology. 2013;52(12):2136–48.

4.	Mikocka-Walus AA, Turnbull DA, Moulding NT, Wilson IG, Andrews JM, Holtmann GJ. Controversies surrounding the comorbidity of depression and anxiety in inflammatory bowel disease patients: A literature review. Inflamm Bowel Dis. 2007;13(2):225–34.

5.	Hanlon P, Hannigan L, Rodriguez-Perez J, Fischbacher C, Welton NJ, Dias S, et al. Representation of people with comorbidity and multimorbidity in clinical trials of novel drug therapies: an individual-level participant data analysis. BMC Med. 2019 Dec 12;17(1):201.

# Supplementary tables

| **Drug** | **WHO-ATC level 5 code** | **ATC-5 class description** | **ATC-7 code** | **N trials** | **Total N participants** |
| --- | --- | --- | --- | --- | --- |
| metformin | A10BA | biguanide | A10BA02 | 4 | 1220 |
| glipizide | A10BB | A10BB Sulfonylureas | A10BB07 | 1 | 700 |
| glimepiride | A10BB | A10BB Sulfonylureas | A10BB12 | 2 | 618 |
| rivoglitazone | A10BG | thiazolidinedione | A10BG_2 | 1 | 910 |
| rosiglitazone | A10BG | thiazolidinedione | A10BG02 | 4 | 2134 |
| pioglitazone | A10BG | thiazolidinedione | A10BG03 | 2 | 948 |
| sitagliptin | A10BH | dpp-4 inhibitor | A10BH01 | 20 | 23766 |
| vildagliptin | A10BH | dpp-4 inhibitor | A10BH02 | 16 | 11158 |
| saxagliptin | A10BH | dpp-4 inhibitor | A10BH03 | 11 | 24404 |
| alogliptin | A10BH | dpp-4 inhibitor | A10BH04 | 5 | 6944 |
| linagliptin | A10BH | dpp-4 inhibitor | A10BH05 | 13 | 14452 |
| gemigliptin | A10BH | dpp-4 inhibitor | A10BH06 | 1 | 288 |
| taspoglutide | A10BJ | glp-1 receptor agonist | A10BJ_3 | 1 | 332 |
| exenatide | A10BJ | glp-1 receptor agonist | A10BJ01 | 7 | 20402 |
| liraglutide | A10BJ | glp-1 receptor agonist | A10BJ02 | 8 | 12534 |
| lixisenatide | A10BJ | glp-1 receptor agonist | A10BJ03 | 13 | 6058 |
| albiglutide | A10BJ | glp-1 receptor agonist | A10BJ04 | 6 | 11142 |
| dulaglutide | A10BJ | glp-1 receptor agonist | A10BJ05 | 4 | 10504 |
| dapagliflozin | A10BK | A10BK Sodium-glucose co-transporter 2 (SGLT2) inhibitors | A10BK01 | 22 | 32198 |
| canagliflozin | A10BK | A10BK Sodium-glucose co-transporter 2 (SGLT2) inhibitors | A10BK02 | 9 | 15724 |
| empagliflozin | A10BK | A10BK Sodium-glucose co-transporter 2 (SGLT2) inhibitors | A10BK03 | 8 | 8390 |
| repaglinide | A10BX | glinide | A10BX02 | 1 | 324 |
| nateglinide | A10BX | glinide | A10BX03 | 1 | 4652 |
| mitiglinide | A10BX | glinide | A10BX08 | 1 | 244 |

sTable 1. Classification of drugs from the diabetes trial-set into classes based on WHO-ATC-5

| **Trial ID** | **WHO-ATC level 5 code (class)** | **Drug** | **Number of participants per experimental group in trial** | **Simulated covariate-treatment interaction effect estimate** | **Standard error of interaction effect estimate (derived^1^)** |
| --- | --- | --- | --- | --- | --- |
| NCT00819741 | A10BA | metformin | 216 | 0.08 | 0.19 |
| NCT01512979 | A10BA | metformin | 158 | 0.07 | 0.23 |
| NCT01545388 | A10BA | metformin | 112 | 0.07 | 0.27 |
| NCT02068443 | A10BA | metformin | 124 | 0.13 | 0.25 |
| NCT00131664 | A10BB | glimepiride | 130 | -0.14 | 0.25 |
| NCT01459809 | A10BB | glimepiride | 179 | -0.11 | 0.21 |
| NCT00086515 | A10BB | glipizide | 350 | -0.19 | 0.15 |
| NCT00094757 | A10BG | pioglitazone | 173 | -0.26 | 0.22 |
| NCT00220961 | A10BG | pioglitazone | 301 | -0.23 | 0.16 |
| NCT00484198 | A10BG | rivoglitazone | 455 | -0.10 | 0.13 |
| NCT00241605 | A10BG | rosiglitazone | 300 | 0.05 | 0.16 |
| NCT00359112 | A10BG | rosiglitazone | 272 | 0.03 | 0.17 |
| NCT00386100 | A10BG | rosiglitazone | 344 | -0.03 | 0.15 |
| NCT00499707 | A10BG | rosiglitazone | 151 | -0.05 | 0.23 |
| NCT00286494 | A10BH | alogliptin | 164 | -0.19 | 0.22 |
| NCT00432276 | A10BH | alogliptin | 401 | -0.34 | 0.14 |
| NCT00968708 | A10BH | alogliptin | 2690 | -0.20 | 0.05 |
| NCT01318070 | A10BH | alogliptin | 113 | -0.24 | 0.27 |
| NCT01318083 | A10BH | alogliptin | 104 | -0.26 | 0.28 |
| NCT01787396 | A10BH | gemigliptin | 144 | -0.10 | 0.24 |
| … |  |  |  |  |  |

sTable 2. Example dataset from simulation (abbreviated to 20 rows)

Notes: ^1^ The standard error of the interaction effect estimate for each trial was derived by assuming a SD of 1 for the main effect in a trial, and using the number of participants per group in each trial to calculate standard errors for subgroups with and without a hypothetical comorbidity (prevalence set at 0.2), which were combined to give the overall variance of the interaction estimate

| **Scenario** | |  | **Single-drug model** | | **Full model** | |
| --- | --- | --- | --- | --- | --- | --- |
| **Level(s)** | **Variation** | **Performance measure** | **Estimate** | **MCSE** | **Estimate** | **MCSE** |
|  |  |  |  |  |  |  |
| All | Low | Bias | 0.013 | 0.000 | 0.001 | 0.000 |
| All | Low | MSE | 0.003 | 0.000 | 0.003 | 0.000 |
| All | Low | RMSE | 0.058 | 0.000 | 0.056 | 0.000 |
| All | Low | Rel. prec. | ⁃ | ⁃ | 122.727 | 1.868 |
| All | Low | Coverage | 0.989 | 0.001 | 0.889 | 0.002 |
| All | Medium | Bias | 0.013 | 0.001 | 0.000 | 0.001 |
| All | Medium | MSE | 0.025 | 0.000 | 0.028 | 0.000 |
| All | Medium | RMSE | 0.160 | 0.001 | 0.167 | 0.001 |
| All | Medium | Rel. prec. | ⁃ | ⁃ | 20.311 | 0.543 |
| All | Medium | Coverage | 0.855 | 0.002 | 0.719 | 0.003 |
| All | High | Bias | 0.010 | 0.002 | -0.003 | 0.002 |
| All | High | MSE | 0.069 | 0.001 | 0.077 | 0.001 |
| All | High | RMSE | 0.263 | 0.001 | 0.277 | 0.001 |
| All | High | Rel. prec. | ⁃ | ⁃ | 7.028 | 0.345 |
| All | High | Coverage | 0.783 | 0.003 | 0.651 | 0.003 |
|  |  |  |  |  |  |  |
| Trial | Medium | Bias | 0.012 | 0.001 | 0.000 | 0.000 |
| Trial | Medium | MSE | 0.009 | 0.000 | 0.005 | 0.000 |
| Trial | Medium | RMSE | 0.093 | 0.000 | 0.069 | 0.000 |
| Trial | Medium | Rel. prec. | ⁃ | ⁃ | 134.132 | 1.922 |
| Trial | Medium | Coverage | 0.977 | 0.001 | 0.918 | 0.002 |
| Trial | High | Bias | 0.014 | 0.001 | 0.002 | 0.001 |
| Trial | High | MSE | 0.018 | 0.000 | 0.007 | 0.000 |
| Trial | High | RMSE | 0.134 | 0.001 | 0.081 | 0.000 |
| Trial | High | Rel. prec. | ⁃ | ⁃ | 221.363 | 2.696 |
| Trial | High | Coverage | 0.968 | 0.001 | 0.950 | 0.001 |
| Drug | Medium | Bias | 0.013 | 0.001 | 0.001 | 0.001 |
| Drug | Medium | MSE | 0.004 | 0.000 | 0.008 | 0.000 |
| Drug | Medium | RMSE | 0.065 | 0.000 | 0.090 | 0.001 |
| Drug | Medium | Rel. prec. | ⁃ | ⁃ | 56.857 | 0.797 |
| Drug | Medium | Coverage | 0.989 | 0.001 | 0.938 | 0.002 |
| Drug | High | Bias | 0.013 | 0.001 | 0.000 | 0.001 |
| Drug | High | MSE | 0.006 | 0.000 | 0.011 | 0.000 |
| Drug | High | RMSE | 0.077 | 0.000 | 0.107 | 0.001 |
| Drug | High | Rel. prec. | ⁃ | ⁃ | 22.996 | 0.399 |
| Drug | High | Coverage | 0.989 | 0.001 | 0.953 | 0.001 |
| Class | Medium | Bias | 0.014 | 0.001 | 0.001 | 0.001 |
| Class | Medium | MSE | 0.019 | 0.000 | 0.019 | 0.000 |
| Class | Medium | RMSE | 0.138 | 0.001 | 0.138 | 0.001 |
| Class | Medium | Rel. prec. | ⁃ | ⁃ | 12.471 | 0.544 |
| Class | Medium | Coverage | 0.846 | 0.002 | 0.606 | 0.003 |
| Class | High | Bias | 0.010 | 0.001 | -0.003 | 0.002 |
| Class | High | MSE | 0.052 | 0.001 | 0.058 | 0.001 |
| Class | High | RMSE | 0.229 | 0.001 | 0.241 | 0.001 |
| Class | High | Rel. prec. | ⁃ | ⁃ | -6.145 | 0.322 |
| Class | High | Coverage | 0.737 | 0.003 | 0.447 | 0.003 |
|  |  |  |  |  |  |  |

sTable 3. Summary of performance measures for full and single drug only models across all simulated datasets for different scenarios – **LOW SIMULATED COMORBIDITY PREVALENCE (10%)**

*Note – See Data generation procedure in Methods for full definition of scenarios; MSE =mean squared error; RMSE=root mean squared error; Rel. precision = % change in precision for full vs. drug model; Coverage = proportion of 95% credible intervals containing true effect; MCSE = Monte Carlo standard errors; RMSE estimates and corresponding MCSEs are not calculated by default in the rsimsum package and so are instead derived, with the MCSE approximated using the delta method, i.e.:*

${SE}_{RMSE}= \sqrt{\frac{Var\left( \sqrt{MSE} \right)}{n}} \approx\sqrt{\frac{{n\left( {SE}_{MSE} \right)}^{2}}{4n \times\hat{MSE}}}= \frac{{SE}_{MSE}}{2 \times\surd\hat{MSE}}$*:*

| **Scenario** | |  | **Single-drug model** | | **Full model** | |
| --- | --- | --- | --- | --- | --- | --- |
| **Level(s)** | **Variation** | **Performance measure** | **Estimate** | **MCSE** | **Estimate** | **MCSE** |
|  |  |  |  |  |  |  |
| All | Low | Bias | 0.013 | 0.000 | 0.001 | 0.000 |
| All | Low | MSE | 0.003 | 0.000 | 0.003 | 0.000 |
| All | Low | RMSE | 0.057 | 0.000 | 0.056 | 0.000 |
| All | Low | Rel. prec. | ⁃ | ⁃ | 65.898 | 1.127 |
| All | Low | Coverage | 0.943 | 0.001 | 0.821 | 0.002 |
| All | Medium | Bias | 0.012 | 0.001 | 0.000 | 0.001 |
| All | Medium | MSE | 0.025 | 0.000 | 0.027 | 0.000 |
| All | Medium | RMSE | 0.159 | 0.001 | 0.165 | 0.001 |
| All | Medium | Rel. prec. | ⁃ | ⁃ | 9.408 | 0.370 |
| All | Medium | Coverage | 0.783 | 0.003 | 0.647 | 0.003 |
| All | High | Bias | 0.010 | 0.002 | -0.003 | 0.002 |
| All | High | MSE | 0.069 | 0.001 | 0.076 | 0.001 |
| All | High | RMSE | 0.263 | 0.001 | 0.276 | 0.001 |
| All | High | Rel. prec. | ⁃ | ⁃ | 2.398 | 0.261 |
| All | High | Coverage | 0.748 | 0.003 | 0.609 | 0.003 |
|  |  |  |  |  |  |  |
| Trial | Medium | Bias | 0.012 | 0.001 | 0.000 | 0.000 |
| Trial | Medium | MSE | 0.008 | 0.000 | 0.004 | 0.000 |
| Trial | Medium | RMSE | 0.090 | 0.000 | 0.067 | 0.000 |
| Trial | Medium | Rel. prec. | ⁃ | ⁃ | 129.466 | 1.703 |
| Trial | Medium | Coverage | 0.944 | 0.001 | 0.884 | 0.002 |
| Trial | High | Bias | 0.013 | 0.001 | 0.001 | 0.000 |
| Trial | High | MSE | 0.017 | 0.000 | 0.006 | 0.000 |
| Trial | High | RMSE | 0.131 | 0.001 | 0.075 | 0.000 |
| Trial | High | Rel. prec. | ⁃ | ⁃ | 263.698 | 2.887 |
| Trial | High | Coverage | 0.955 | 0.001 | 0.942 | 0.002 |
| Drug | Medium | Bias | 0.013 | 0.001 | 0.001 | 0.001 |
| Drug | Medium | MSE | 0.004 | 0.000 | 0.006 | 0.000 |
| Drug | Medium | RMSE | 0.064 | 0.000 | 0.076 | 0.000 |
| Drug | Medium | Rel. prec. | ⁃ | ⁃ | 21.332 | 0.425 |
| Drug | Medium | Coverage | 0.943 | 0.001 | 0.860 | 0.002 |
| Drug | High | Bias | 0.012 | 0.002 | 0.000 | 0.001 |
| Drug | High | MSE | 0.006 | 0.000 | 0.006 | 0.000 |
| Drug | High | RMSE | 0.076 | 0.000 | 0.080 | 0.001 |
| Drug | High | Rel. prec. | ⁃ | ⁃ | 2.542 | 0.215 |
| Drug | High | Coverage | 0.943 | 0.001 | 0.867 | 0.002 |
| Class | Medium | Bias | 0.013 | 0.001 | 0.001 | 0.001 |
| Class | Medium | MSE | 0.019 | 0.000 | 0.021 | 0.000 |
| Class | Medium | RMSE | 0.139 | 0.001 | 0.145 | 0.001 |
| Class | Medium | Rel. prec. | ⁃ | ⁃ | -1.838 | 0.384 |
| Class | Medium | Coverage | 0.739 | 0.003 | 0.494 | 0.003 |
| Class | High | Bias | 0.010 | 0.002 | -0.003 | 0.002 |
| Class | High | MSE | 0.053 | 0.001 | 0.062 | 0.001 |
| Class | High | RMSE | 0.230 | 0.001 | 0.249 | 0.001 |
| Class | High | Rel. prec. | ⁃ | ⁃ | -12.440 | 0.263 |
| Class | High | Coverage | 0.635 | 0.003 | 0.333 | 0.003 |
|  |  |  |  |  |  |  |

sTable 4 Summary of performance measures for full and single drug only models across all simulated datasets for different scenarios – **HIGH SIMULATED COMORBIDITY PREVALENCE (50%)**

*Note – See Data generation procedure in Methods for full definition of scenarios; MSE =mean squared error; RMSE=root mean squared error; Rel. precision = % change in precision for full vs. drug model; Coverage = proportion of 95% credible intervals containing true effect; MCSE = Monte Carlo standard errors; RMSE estimates and corresponding MCSEs are not calculated by default in the rsimsum package and so are instead derived, with the MCSE approximated using the delta method, i.e.:*

${SE}_{RMSE}= \sqrt{\frac{Var\left( \sqrt{MSE} \right)}{n}} \approx\sqrt{\frac{{n\left( {SE}_{MSE} \right)}^{2}}{4n \times\hat{MSE}}}= \frac{{SE}_{MSE}}{2 \times\surd\hat{MSE}}$*:*

# Supplementary figures

sFigure 1 Summary of relative precision of drug level comorbidity-treatment interaction effects in full vs. single drug model as a function of drug class– **LOW SIMULATED COMORBIDITY PREVALENCE (10%)**

*Note – error bars show Monte-Carlo standard errors; information on the number and size of trials for each drug class is found in sTable 1*

sFigure 2 Summary of relative precision of drug level comorbidity-treatment interaction effects in full vs. single drug model as a function of drug class– **HIGH SIMULATED COMORBIDITY PREVALENCE (50%)**

*Note – error bars show Monte-Carlo standard errors; information on the number and size of trials for each drug class is found in sTable 1*

sFigure 3 Illustration of the impact of increased precision in the full model: summarising the proportion of all datasets with “true” effects in the three main scenarios in which credible intervals for the interaction effect estimate for each drug excluded zero (i.e., no interaction) in i) both models; ii) the single drug model only; and iii) the full model only, alongside enrollment information – **LOW SIMULATED COMORBIDITY PREVALENCE (10%)**

sFigure 4 Illustration of the impact of increased precision in the full model: summarising the proportion of all datasets with “true” effects in the three main scenarios in which credible intervals for the interaction effect estimate for each drug excluded zero (i.e., no interaction) in i) both models; ii) the single drug model only; and iii) the full model only, alongside enrollment information – **HIGH SIMULATED COMORBIDITY PREVALENCE (50%)**
